# Pharmacokinetics and hematologic toxicity of linezolid in children: a prospective, two-center cohort study

**DOI:** 10.1128/aac.00294-25

**Published:** 2025-07-23

**Authors:** Lvchang Zhu, Xinxin Zeng, Yuhang Wu, Xuben Yu, Shanshan Xu, Qiuxia Wang, Xiaoshan Zhang, Xi Zhang, Qiang Shu, Zihao Yang, Lisu Huang

**Affiliations:** 1 Pediatric Intensive Care Unit, Children’s Hospital, Zhejiang University School of Medicine, National Clinical Research Center for Child Health 666702, Hangzhou, Zhejiang, China; 2 Department of Infectious Diseases, Children’s Hospital, Zhejiang University School of Medicine, National Clinical Research Center for Child Health 666702, Hangzhou, Zhejiang, China; 3 Department of Pharmacy, The First Affiliated Hospital of Wenzhou Medical University 89657, Wenzhou, Zhejiang, China; 4 Department of Infectious Disease, Xinhua Hospital Affiliated to Shanghai Jiao Tong University School of Medicine 91603 , Shanghai, China; 5 Clinical Research Unit, Xinhua Hospital Affiliated to Shanghai Jiao Tong University School of Medicine 91603 , Shanghai, China; 6 Department of Thoracic and Cardiovascular Surgery, Children’s Hospital, Zhejiang University School of Medicine, National Clinical Research Center for Child Health 666702, Hangzhou, Zhejiang, China; University of Pittsburgh School of Medicine, Pittsburgh, Pennsylvania, USA

**Keywords:** hematologic toxicity, pharmacokinetics, linezolid, anti-cancer chemotherapy, therapeutic drug monitoring, pediatric

## Abstract

Despite the widespread pediatric use of linezolid, data on its hematologic toxicity—particularly among children exposed to anticancer chemotherapy—remain limited and inconsistent. This study aimed to evaluate linezolid-induced hematotoxicity through pharmacokinetic analysis, with an emphasis on chemotherapy-exposed pediatric patients. This dual-center prospective study assessed linezolid pharmacokinetics and clinical profiles in chemotherapy-stratified pediatric cohorts, examining associations with hematologic toxicity. Among 229 pediatric patients (65 with cancer), hematologic toxicity occurred in 43.2%, with significantly higher risks of leukopenia (hazard ratio [HR] 20.29, 95% confidence interval [CI]: 3.98–103.38), neutropenia (HR 2.60, 95% CI: 1.00–6.77), and thrombocytopenia events (HR 7.08, 95% CI: 2.19–22.92) in cancer patients. Median linezolid trough and peak concentrations were 2.60 mg/L (interquartile range [IQR] 1.42–4.06) and 12.00 mg/L (IQR 9.5–14.19), respectively. Among cancer patients, trough concentrations above 7 mg/L elevated leukopenia (HR 6.33, 95% CI: 1.36–29.42) and anemia event (HR 8.72, 95% CI: 1.98–38.37) risk. Prolonged therapy exceeding 14 days elevated the risk of anemia events (HR 2.17; 95% CI: 1.08–4.35), while durations beyond 28 days also increased the risk of neutropenia events (HR 3.58; 95% CI, 1.37–9.32). At equivalent daily doses, twice-daily dosing resulted in higher peak concentrations (19.65 vs 13.67 mg/L; *P* = 0.020) and a greater incidence of anemia events (62.50% vs 25.00%; *P* = 0.033) compared to thrice-daily regimens. Linezolid frequently causes hematologic toxicity in children, particularly in chemotherapy recipients. Risk is also driven by high concentrations (peak > 15 mg/L, trough > 7 mg/L in cancer patients), prolonged therapy, and twice-daily dosing, necessitating careful monitoring and dose optimization.

## INTRODUCTION

Linezolid, the first oxazolidinone antibiotic, is a potent inhibitor of bacterial protein synthesis with broad activity against aerobic gram-positive pathogens, including methicillin-resistant *Staphylococcus aureus* and vancomycin-resistant *Enterococcus* (1). It is widely used to treat severe infections such as pneumonia, skin and soft tissue infections, bacteremia, and osteomyelitis (2–4). In recent years, pediatric use of linezolid has increased significantly, with a 121% rise reported between 2013 and 2020 (5, 6). This trend highlights the need for a thorough evaluation of its safety profile in children, particularly given the metabolic and pharmacokinetic differences between pediatric and adult populations (7, 8).

Hematologic toxicity, a well-documented adverse effect of linezolid, is thought to result from its inhibition of mitochondrial protein synthesis and bone marrow suppression (9). While short-term treatment (<2 weeks) in adults is associated with a low incidence of hematologic adverse events (9, 10), pediatric studies report widely varying rates, ranging from <1% to 53% (5, 8, 11). This variability may stem from small sample sizes, retrospective study designs, and differences in treatment duration (8, 11). Notably, long-term linezolid use in children is increasingly common, with hematologic adverse event rates ranging from 6.7% to 58.8%, suggesting a potential correlation between treatment duration and toxicity (12–14). However, the relationship remains poorly defined, particularly in high-risk pediatric populations, such as those with hematologic malignancies or undergoing anti-cancer chemotherapy, who often require prolonged linezolid therapy for severe gram-positive infections (15).

Current dosing guidelines for linezolid in children differ by age: those under 12 years are dosed by weight, while those 12 years and older follow adult dosing (16). This discrepancy raises concerns about potential differences in hematologic toxicity between age groups, which have not been thoroughly investigated. Therapeutic drug monitoring (TDM) has been proposed as a strategy to optimize linezolid dosing and minimize toxicity (8), but its use in pediatric patients remains limited. Further research is needed to clarify the pharmacokinetic-pharmacodynamic relationships of linezolid in children and identify predictors of hematologic toxicity.

This study aims to address these gaps by prospectively evaluating the incidence of hematologic adverse events in pediatric patients receiving linezolid, with a focus on those with hematologic malignancies or undergoing anti-cancer chemotherapy, as well as previously healthy children. Additionally, we will measure linezolid concentrations to explore the relationship between pharmacokinetics and hematologic toxicity. By elucidating these factors, we hope to contribute to the safe and effective use of linezolid in pediatric clinical practice.

## MATERIALS AND METHODS

### Study design

This prospective observational study was conducted at two tertiary care centers in China: the Children’s Hospital affiliated with Zhejiang University School of Medicine and Xinhua Hospital affiliated with Shanghai Jiao Tong University School of Medicine. We enrolled pediatric patients (<18 years old) from the intensive care unit and infectious disease department who received at least five doses of linezolid. Patients were stratified into two groups: (i) the cancer group, comprising children with hematologic malignancies or malignant solid tumors who were undergoing or had undergone anti-cancer chemotherapy within 1 month prior to linezolid administration; and (ii) the non-cancer group, comprising previously healthy children. Exclusion criteria included patients without valid blood concentration test samples, those undergoing renal replacement therapy, and those without consent from guardians or the children themselves. The study population included children treated with linezolid between January 2021 and August 2023. Linezolid administration followed clinical guidelines and was adjusted at the discretion of the attending physician. Hematologic adverse events related to linezolid were monitored using clinical laboratory results during the treatment period and for 2 weeks after discontinuation. Blood samples were collected for linezolid concentration measurements.

### Data collection and linezolid concentration measurement

Upon participant consent, baseline demographic and clinical data were collected from hospital records at the initiation of linezolid therapy. These data encompassed age, sex, anthropometric measurements (height and weight), laboratory results, and details regarding linezolid administration. If hematologic parameters were not available on the exact day treatment commenced, values from the immediately preceding or succeeding day were utilized. Hemoglobin levels measured within 10 days post-red blood cell transfusion and platelet counts measured within 5 days post-platelet transfusion were excluded from the statistical analysis of adverse events. Additional clinical information, such as pathogen identification, underlying diagnosis, and treatment outcomes, was also documented.

To evaluate steady-state linezolid concentrations, blood samples were obtained from participants after a minimum of five consecutive doses. Sample collection was timed to coincide with clinically indicated phlebotomy, targeting either peak concentrations (defined as 30 min post-intravenous infusion or 2 hours post-oral administration) or trough concentrations (defined as within 30 min prior to the next scheduled dose). If precise timing for peak or trough sampling was clinically impractical, random timed samples were collected in accordance with the patient’s clinical schedule. For participants who consented to serial pharmacokinetic monitoring, an additional blood sample was typically collected approximately 1 week following the initial sampling. A minimum of one peak or one trough sample was targeted for each participant, with the collection of supplementary samples (random, peak, or trough) pursued when clinically feasible. Linezolid concentrations were measured using high-performance liquid chromatography-tandem mass spectrometry (17), with a lower limit of quantification of 0.1 mg/L. The intra- and inter-day variability of the assay was less than 10%. The precise times of administration and sampling were recorded to estimate linezolid peak blood concentrations (*C*_max_) and linezolid trough blood concentrations (*C*_min_).

### Definition

Adverse events were categorized based on significant deviations from baseline values. Hematologic adverse events related to linezolid therapy included leukopenia, neutropenia, thrombocytopenia, and anemia. Normal lower limits were defined as follows: white blood cell count 4 × 10^9^/L, neutrophil count 1.5 × 10^9^/L, and platelet count 100 × 10^9^/L. Hemoglobin lower limits varied by age: 90 g/L for 1–4 months, 100 g/L for 4–6 months, 110 g/L for 6 months to 6 years, and 120 g/L for children over 6 years (18). Hematologic events were identified as a >25% decrease from baseline values and falling below the age-specific lower limit of normal (19, 20). Adverse event severity was graded using the Common Terminology Criteria for Adverse Events version 5.0, with grade 1 indicating mild events (21). Peripheral blood counts were monitored every 2–4 days during linezolid therapy, with timing adjusted clinically to minimize sampling burden.

### Data analysis

The predicted steady-state *C*_min_ or *C*_max_ for each patient was estimated using the maximum *a posteriori* (MAP) Bayesian estimation method in NONMEM, based on our previously developed pediatric population pharmacokinetic model of linezolid (22), with each patient’s measured concentrations serving as the Bayesian prior information. For statistical analyses, we exclusively used these model-predicted *C*_min_ or *C*_max_ rather than raw measurements.

The incidence of linezolid-related hematologic adverse events was analyzed in both the cancer and non-cancer groups. Stratified analyses were conducted based on age group, treatment duration, and dosing regimen. Additionally, pharmacokinetic (PK) indicators, specifically the *C*_min_ and *C*_max_ of linezolid, were evaluated. Given the differing dosing instructions for children above and below 12 years of age, a gender- and weight-matched analysis was performed in children around 12 years old to compare the incidence of hematologic adverse events under two dosing regimens: 600 mg twice daily and 10 mg/kg of body weight three times daily.

Data are presented as frequencies (percentages) for categorical variables, medians with interquartile ranges (IQR), and mean ± standard deviation (SD) for continuous variables. Continuous variables were compared using Student’s *t*-test, while categorical variables were assessed using chi-square or Fisher’s exact tests, as appropriate. Time-to-event data for adverse events were analyzed using Kaplan-Meier curves, stratified by categorical variables. Multivariate Cox proportional hazards regression models were used to calculate hazard ratios.

All statistical tests were two-tailed, with a *P*-value of <0.05 considered statistically significant. Analyses were performed using EmpowerStats (version 4.1, USA) and R software (version 4.1.1, New Zealand).

## RESULTS

### Study population characteristics

Out of 244 children who received at least five doses of linezolid, 229 were included in the final analysis (Fig. S1), comprising 65 in the cancer group and 164 in the non-cancer group. The cohort was predominantly male (56.77%), with a median age of 5.80 years (IQR: 1.14–9.45). The most common infection sites were central nervous system (22.71%), osteoarticular (14.41%), skin and soft tissue (16.59%), intra-abdominal (12.23%), and pulmonary (13.54%). Gram-positive bacteria were identified in 34.06% of patients. The median treatment duration was 13 days (IQR: 7–24), with linezolid dosing adhering to guidelines: a median dose of 10.00 mg/kg of body weight (IQR: 9.50–10.00) and a daily dose of 29.66 mg/kg of body weight (IQR: 27.93–30.00). Hematologic abnormalities were significantly more prevalent in the cancer group, including leukopenia (29.23% vs 0.61%), neutropenia (33.85% vs 3.05%), anemia (75.38% vs 24.39%), and thrombocytopenia (52.31% vs 6.17%). Liver function tests and blood urea nitrogen (BUN) levels were comparable between groups, while serum creatinine was modestly higher in the cancer group but remained within normal limits (Table 1).

**TABLE 1:** Characteristics of children at the initiation of linezolid treatment^*a*^

Characteristics	All patients (*n* = 229)	Non-cancer (*n* = 164)	Cancer (*n* = 65)
Age (years), median (IQR)	5.80 (1.14–9.45)	4.54 (0.68–8.97)	8.34 (4.32–11.57)
Male, *n* (%)	130 (56.77)	93 (56.71)	37 (56.92)
Site of infection, *n* (%)			
Bacterial meningitis	52 (22.71)	42 (25.61)	10 (15.38)
Osteoarthritis	33 (14.41)	28 (17.07)	5 (7.69)
Skin and soft tissue	38 (16.59)	29 (17.68)	9 (13.85)
Intra-abdominal	28 (12.23)	19 (11.59)	9 (13.85)
Lung	31 (13.54)	25 (15.24)	6 (9.23)
Undefined	47 (20.52)	21 (12.80)	26 (40.00)
Pathogen, *n* (%)			
Confirmed	78 (34.06)	59 (35.98)	19 (29.23)
Unconfirmed	151 (65.94)	105 (64.02)	46 (70.77)
Baseline laboratory data			
Leukopenia, *n* (%)	20 (8.73)	1 (0.61)	19 (29.23)
Neutropenia, *n* (%)	27 (11.79)	5 (3.05)	22 (33.85)
Anemia, *n* (%)	89 (38.86)	40 (24.39)	49 (75.38)
Thrombocytopenia, *n* (%)	44 (19.38)	10 (6.17)	34 (52.31)
ALT (IU/L), median (IQR)	21.00 (13.00–35.00)	21.00 (13.23–34.25)	21.10 (12.00–38.50)
TBil (mmol/L), median (IQR)	7.80 (5.00–13.55)	7.40 (5.00–13.25)	9.20 (5.70–14.85)
BUN (mmol/L), median (IQR)	3.80 (2.80–5.16)	3.69 (2.73–4.93)	4.39 (3.02–5.95)
SCr (μmol/L), median (IQR)	24.00 (18.00–36.00)	23.00 (18.00–34.00)	31.00 (21.75–45.00)
Treatment of LZD, median (IQR)			
Duration of LZD (days)	13.00 (7.00–24.00)	14.00 (7.00–26.00)	12.00 (8.00–18.00)
Each dose of LZD (mg/kg of body weight)	10.00 (9.50–10.00)	10.00 (9.50–10.00)	9.94 (9.49–10.38)
Daily dose of LZD (mg/kg of body weight)	29.66 (27.93–30.00)	30.00 (28.24–30.00)	29.19 (26.12–30.00)
Concentration, median (IQR)			
*C*_min_ (mg/L)	2.60 (1.42–4.06)	2.58 (1.40–3.95)	2.61 (1.46–4.18)
*C*_max_ (mg/L)	12.00 (9.5–14.19)	11.56 (9.30–13.99)	13.24 (10.53–15.18)

^*a*^ ALT, alanine aminotransferase; TBil, total bilirubin; SCr, serum creatinine; LZD, linezolid; *C*_max_, peak linezolid blood concentration; and *C*_min_, trough linezolid blood concentration.

### Hematologic toxicity in cancer and non-cancer patients

**Fig 1:**
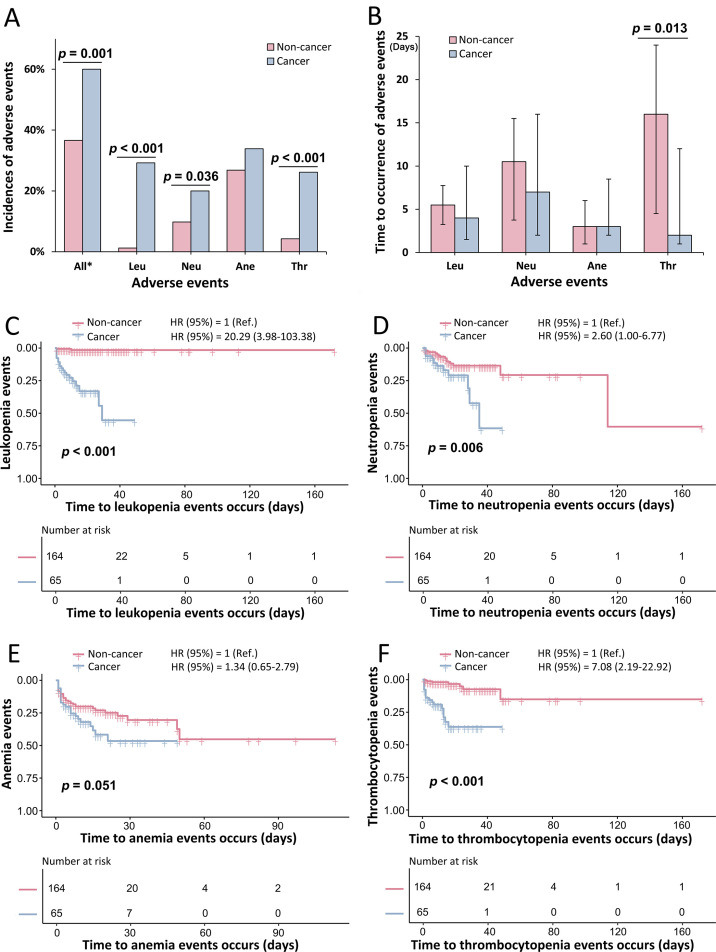
Linezolid-associated hematologic adverse events stratified by anti-cancer chemotherapy status. Cancer group exhibits significantly higher rates of leukopenia, neutropenia, and thrombocytopenia events (**A**), with thrombocytopenia events occurring earlier (**B**). Kaplan-Meier curves indicate that the cancer group is at a greater risk for leukopenia, neutropenia, and thrombocytopenia events; a similar trend is observed for anemia events, although the difference does not reach statistical significance (**C–F**). All *, all hematologic adverse events. Leu, leukopenia adverse events; Neu, neutropenia adverse events; Ane, anemia adverse events; and Thr, thrombocytopenia adverse events. HR: adjusted age, gender, BMI, baseline blood cell count (declined or not), site of infection, and pathogen confirmed (with or without).

Two patients maintained concurrent anticancer chemotherapy (homoharringtonine and cyclosporine) during linezolid treatment, while others had discontinued such therapy. Notably, neither case developed hematological adverse events. The incidence of hematologic adverse events was significantly higher in the cancer group than in the non-cancer group (60.00% vs 36.59%, *P* = 0.001) (Table S1). Kaplan-Meier analysis demonstrated significantly higher incidence rates of leukopenia, neutropenia, and thrombocytopenia adverse events in cancer patients compared to the non-cancer group (Fig. 1C, D and F). ‌Multivariate Cox regression analysis with stepwise adjustment for age, gender, BMI, baseline blood cell counts, infection site, and pathogen identification status demonstrated significantly higher hematologic toxicity risks in cancer patients compared to non-cancer controls. Specifically, the cancer group showed markedly increased hazards for leukopenia (hazard ratio [HR] 20.29, 95% confidence interval [CI]: 3.98–103.38; incidence 29.23% vs 1.22%), neutropenia (HR 2.60, 95% CI: 1.00–6.77; 20.00% vs 9.76%), and thrombocytopenia events (HR 7.08, 95% CI: 2.19–22.92; 26.15% vs 4.27%) (Fig. 1; Table S2). Propensity score-matched analysis‌ (30 cancer patients vs 60 non-cancer patients) further demonstrated that the cancer group exhibited significantly higher frequencies of ‌leukopenia events‌ (30.0% vs 1.7%, *P* < 0.001) and ‌thrombocytopenia events‌ (23.3% vs 1.7%, *P* = 0.003) compared to the non-cancer group (‌Table 2). A similar numerical trend was observed for ‌neutropenia events‌, though this did not reach statistical significance (**‌**Table 2). Anemia incidence, affecting 28.82% of patients overall, did not differ significantly between groups. Most adverse events were grade 1; however, grades 2–4 anemia and thrombocytopenia occurred in 21.54% and 20.00% of cancer patients, respectively (Table S1).

**TABLE 2:** Propensity score-matched comparison of linezolid-induced hematologic toxicity in cancer vs non-cancer cohorts^*a*^

Variables	Non-cancer *n* = 60	Cancer *n* = 30	*P-*value
Age (years), mean ± 2SD	5.06 ± 4.33	7.55 ± 4.95	0.016
Gender (female), *n* (%)	27 (45.00)	12 (40.00)	0.822
Clinical features			
ALT (IU/L), mean ± 2SD	27.98 ± 40.91	39.72 ± 44.72	0.24
TBil (mmol/L), mean ± 2SD	14.27 ± 34.49	31.82 ± 114.52	0.3152
BUN (mmol/L), mean ± 2SD	4.24 ± 2.95	4.06 ± 1.89	0.7765
CrCL (mL/min), mean ± 2SD	185.61 ± 79.68	191.69 ± 88.37	0.7651
Confirmed pathogen, *n* (%)	26 (43.3)	9 (30.0)	0.320
Site of infection^*b*^			0.132
Therapy duration (days), mean ± 2SD	25.75 ± 26.39	19.47 ± 11.83	0.2181
*C*_min_ (mg/L), mean ± 2SD	3.89 ± 10.44	3.18 ± 2.51	0.7162
*C*_max_ (mg/L), mean ± 2SD	12.80 ± 11.18	13.14 ± 4.59	0.8727
Baseline data, *n* (%)			
Leukopenia	0	0	NA^*c*^
Neutropenia	2 (3.3)	1 (3.3)	1.000
Anemia	34 (56.7)	17 (56.7)	1.000
Thrombocytopenia	2 (3.3)	1 (3.3)	1.000
Adverse events, *n* (%)			
Leukopenia events	1 (1.7)	9 (30.0)	<0.001
Neutropenia events	6 (10.0)	7 (23.3)	0.168
Anemia events	16 (26.7)	7 (23.3)	0.932
Thrombocytopenia events	1 (1.7)	7 (23.3)	0.003

^*a*^ ALT, alanine aminotransferase; TBil, total bilirubin; CrCL, creatinine clearance rate; LZD, linezolid; *C*_max_, peak linezolid blood concentration; and *C*_min_, trough linezolid blood concentration.

^*b*^ Include bacterial meningitis, osteoarthritis, skin and soft tissue, intra-abdominal, lung, and undefined.

^*c*^ Not applicable. No events in either group, so no statistical comparison was performed.

Regarding the onset of adverse events, neutropenia and thrombocytopenia events in the non-cancer group occurred later (median: 10.50 days [IQR: 3.75–15.50] and 16.00 days [IQR: 4.50–24.00], respectively), while leukopenia and anemia events appeared earlier (5.50 days [IQR: 3.25–7.75] and 3.50 days [IQR: 1.00–8.50], respectively). In the cancer group, anemia and thrombocytopenia events occurred earlier (2.00 days [IQR: 2.00–9.25] and 2.00 days [IQR: 1.00–12.00], respectively) (Table S1).

### Linezolid blood concentration and hematologic toxicity

We analyzed 422 blood samples for linezolid concentrations, including 116 troughs, 96 peaks, and 210 random concentrations. The estimated PK parameters for each patient, including *C*_max_, *C*_min_, individual clearance, and others, are presented in an Excel spreadsheet (Table S3). Steady-state linezolid concentrations were comparable between the cancer and non-cancer groups, with a median *C*_min_ of 2.60 mg/L (IQR: 1.42–4.06) and *C*_max_ of 12.00 mg/L (IQR: 9.5–14.19) (Table 1).

Patients with *C*_max_ > 15 mg/L (approximating the 75th percentile in the general population) exhibited significantly higher incidences of leukopenia events (17.02% vs 7.14%, *P* = 0.036), anemia events (38.30% vs 23.63%, *P* = 0.043), and thrombocytopenia events (19.15% vs 8.24%, *P* = 0.030) compared to those with *C*_max_ ≤ 15 mg/L (Fig. S2; Table S4). In the cancer group, anemia event incidence was significantly higher with *C*_max_ > 15 mg/L (55.56% vs 27.66%, *P* = 0.035), while leukopenia and thrombocytopenia events showed similar trends without statistical significance (Fig. S2). Among cancer patients, *C*_min_ > 7 mg/L was associated with a significantly increased incidence of anemia events (80.00% vs 31.67%, *P* = 0.030) (Fig. S2; Table S5). Multivariable Cox regression analysis was performed for cancer patients with stepwise inclusion of covariates (including age, gender, BMI, baseline blood cell counts, and cancer type) revealed that a *C*_min_ > 7 mg/L‌ demonstrated significantly higher risk‌ of both ‌leukopenia events‌ (HR 6.33, 95% CI: 1.36–29.42) and ‌anemia events (HR 8.72, 95% CI: 1.98–38.37) (Table 3).‌

**TABLE 3:** Stepwise multivariable Cox regression analysis stratified by trough concentration in cancer patients^*a*^

Variables, HR (95% CI)	Leukopenia events	Neutropenia events	Anemia events	Thrombocytopenia events
Crude				
*C*_min_ ≤ 7	1	1	1	1
*C*_min_ > 7	3.70 (1.06, 12.92)	1.51 (0.19, 11.97)	3.95 (1.32, 11.87)	2.33 (0.52, 10.41)
Adjusted age, gender, and BMI				
*C*_min_ ≤ 7	1	1	1	1
*C*_min_ > 7	6.29 (1.50, 26.34)	2.01 (0.23, 17.61)	7.94 (1.90, 33.20)	2.31 (0.27, 19.86)
Adjusted age, gender, BMI, baseline blood cell counts,^*b*^ and cancer type^*c*^				
*C*_min_ ≤ 7	1	1	1	1
*C*_min_ > 7	6.33 (1.36, 29.42)	2.35 (0.23, 24.23)	8.72 (1.98, 38.37)	1.75 (0.19, 16.20)

^*a*^ BMI, body mass index.

^*b*^ Declined or not.

^*c*^ Solid malignancies or hematologic malignancies.

### Linezolid dosage and hematologic toxicity

Children receiving ≥11 mg/kg body weight per dose of linezolid had a higher *C*_max_ compared to those receiving <11 mg/kg of body weight (*P* < 0.001), with no significant differences in *C*_min_ or the area under the concentration curve across 24 hours at steady state (AUC_ss,24h_) (Table S6). Higher doses were associated with increased risks of anemia (HR 2.23, 95% CI 1.13–4.40; 52.63% vs 24.29%) and thrombocytopenia events (HR 2.99, 95% CI: 1.11–8.09; 26.32% vs 9.05%), with a non-significant trend for leukopenia events (Fig. S3A through C; Table S6). In the cancer group, the risk of anemia events was also significantly higher with ≥11 mg/kg of body weight per dose (HR 2.86, 95% CI 1.16–7.04; 70.00% vs 29.09%), with similar non-significant trends for leukopenia and thrombocytopenia adverse events (Fig. S3A and D). Neutropenia adverse events showed no differences at both dosages across different populations (Fig. S3A). For total daily dose, exceeding 30 mg/kg of body weight did not significantly impact hematologic adverse events (Table S7).

### Duration of linezolid treatment and hematologic toxicity

**Fig 2:**
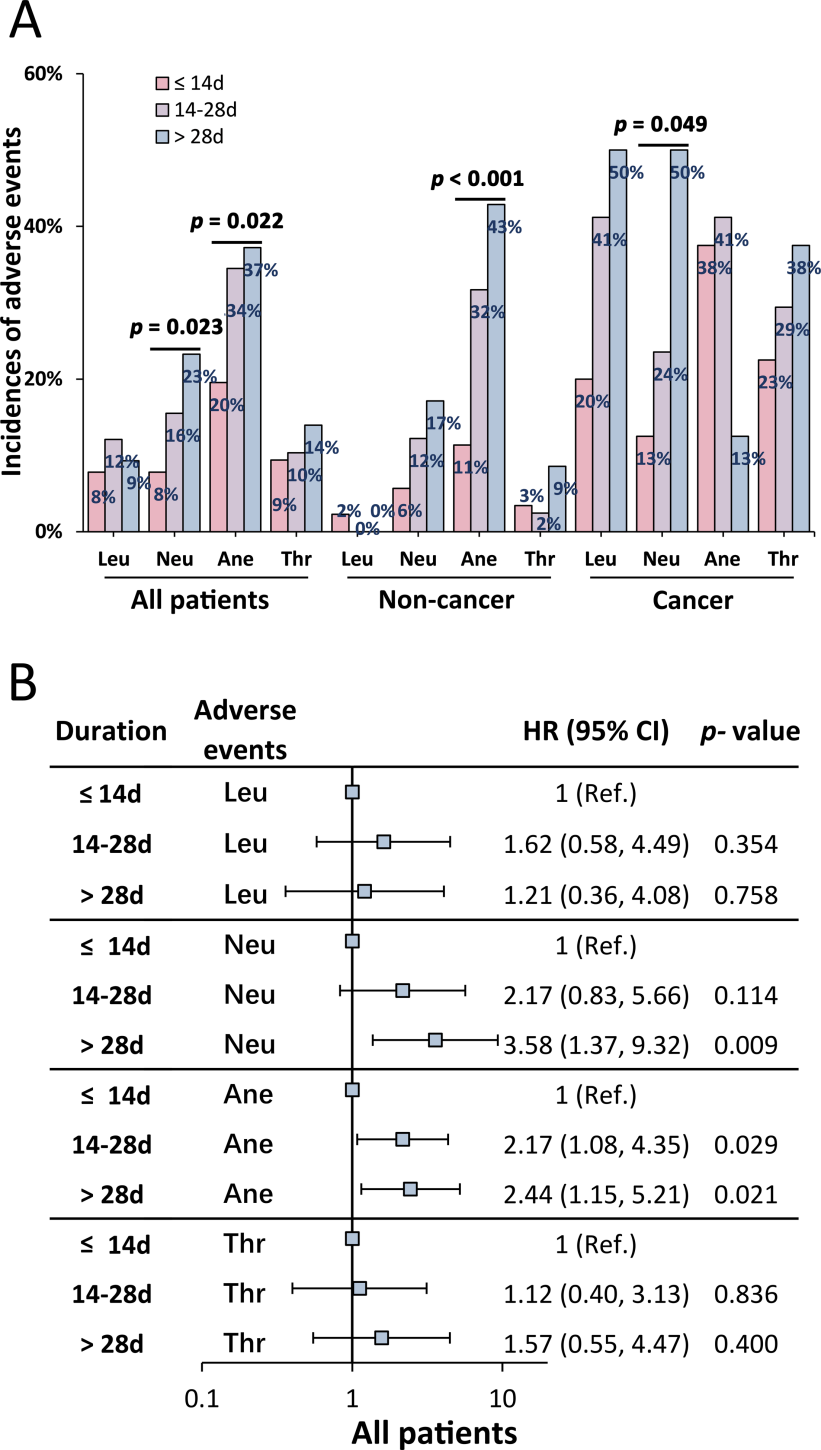
Incidence of linezolid-associated hematologic adverse events stratified by the duration of linezolid therapy. In the overall patient cohort, the incidence of neutropenia and anemia events significantly increased with longer durations of linezolid therapy. Specifically, in the non-cancer group, the incidence of anemia events was significantly higher, whereas in the cancer group, the incidence of neutropenia events was significantly higher with extended linezolid therapy (**A**). Forest plots revealed that the risk of anemia events in the group treated for >14 days to ≤28 days and the risks of anemia and neutropenia events in the group treated for >28 days were elevated compared to the group treated for ≤14 days (**B**). Leu, leukopenia adverse events; Neu, neutropenia adverse events; Ane, anemia adverse events; Thr, thrombocytopenia adverse events; and OR, odds ratio.

Patients were stratified by treatment duration: ≤14, 14–28, and >28 days. Neutropenia and anemia event incidences increased significantly with longer durations (*P* = 0.023 and *P* = 0.022, respectively) (Fig. 2A). Neutropenia event rates were 7.81%, 15.52%, and 23.26% for ≤14, 14–28, and >28 days, respectively. Anemia event incidence was higher in the >28-day (37.21%) and 14–28-day (34.48%) groups compared to the ≤14-day group (19.53%) (Fig. 2A; Table S8). Logistic regression showed higher risks of anemia events in the 14–28 days group (odds ratio [OR] 2.17, 95% CI: 1.08–4.35) and of neutropenia events (OR 3.58, 95% CI: 1.37–9.32) and anemia events (OR 2.44, 95% CI: 1.15–5.21) in the >28 days group compared to ≤14 days (Fig. 2B). Prolonged treatment significantly increased anemia event incidence in the non-cancer group (*P* < 0.001) and neutropenia event incidence in the cancer group (*P* = 0.049). Treatment duration did not significantly affect leukopenia or thrombocytopenia events (Fig. 2; Table S8).

### Age and hematologic toxicity

Analysis across age subgroups (<3, 3–6, 7–11, and ≥12 years) suggested higher incidences of leukopenia, anemia, and thrombocytopenia events in individuals aged ≥12 years, though differences were not statistically significant (Table S9). A gender- and weight-matched analysis of 32 children (16 per group) around 12 years revealed no significant differences in baseline characteristics, including weight, treatment duration, daily dose, or pre-linezolid hematologic parameters (Table 4). However, the 600 mg twice daily group received higher per-dose amounts (11.80 mg/kg of body weight [IQR: 10.79–13.45] vs 9.80 mg/kg of body weight [IQR: 9.32–10.00], *P* < 0.001) and had higher *C*_max_ (19.65 mg/L [IQR: 15.64–21.70] vs 13.67 mg/L [IQR: 8.76–16.37], *P* = 0.020) compared to the 10 mg/kg of body weight three times daily group (Table 4). Anemia event incidence was significantly higher in the 600 mg twice daily group (62.50% vs 25.00% *p* = 0.033) than in the 10 mg/kg of body weight three times daily group, with non-significant trends for leukopenia and thrombocytopenia. Neutropenia rates were comparable (Table 4).

**TABLE 4:** Comparison of adverse events of linezolid between weight-matched children^*a*^

Matched patients	Dosage and administration		*P-*value
	10 mg/kg of body weight, tid (*n* = 16)	600 mg, bid (*n* = 16)	
Characteristics			
Weight (kg), median (IQR)	45.45 (39.90–52.00)	46.95 (42.75–52.12)	0.712
Age (years), median (IQR)	10.47 (9.46–11.38)	13.36 (12.98–14.21)	<0.001
Duration of LZD (days), median (IQR)	8.50 (7.75–24.75)	14.00 (8.00–18.50)	0.575
Per dose (mg/kg of body weight), median (IQR)	9.80 (9.32–10.00)	11.80 (10.79–13.45)	<0.001
Daily dose (mg/kg of body weight), median (IQR)	28.80 (25.53–29.78)	25.56 (23.02–28.37)	0.700
*C*_min_ (mg/L), median (IQR)	3.75 (1.90–4.13)	2.68 (1.12–4.09)	0.803
*C*_max_ (mg/L), median (IQR)	13.67 (8.76–16.37)	19.65 (15.64–21.70)	0.020
Leukopenia, *n* (%)	2 (12.50)	2 (12.50)	1.000
Neutropenia, *n* (%)	1 (6.25)	2 (12.50)	0.544
Anemia, *n* (%)	5 (31.25)	7 (46.67)	0.379
Thrombocytopenia, *n* (%)	3 (18.75)	4 (26.67)	0.598
Adverse events, *n* (%)			
Leukopenia events	2 (12.50)	5 (31.25)	0.200
Neutropenia events	2 (12.50)	2 (12.50)	1.000
Anemia events	4 (25.00)	10 (62.50)	0.033
Thrombocytopenia events	1 (6.25)	5 (31.25)	0.070

^*a*^ Tid, *ter in die*; bid, *bis in die*; LZD, linezolid; *C*_min_, trough linezolid blood concentration; and *C*_max_, peak linezolid blood concentration.

### Decision tree analysis of hematologic adverse events

**Fig 3:**
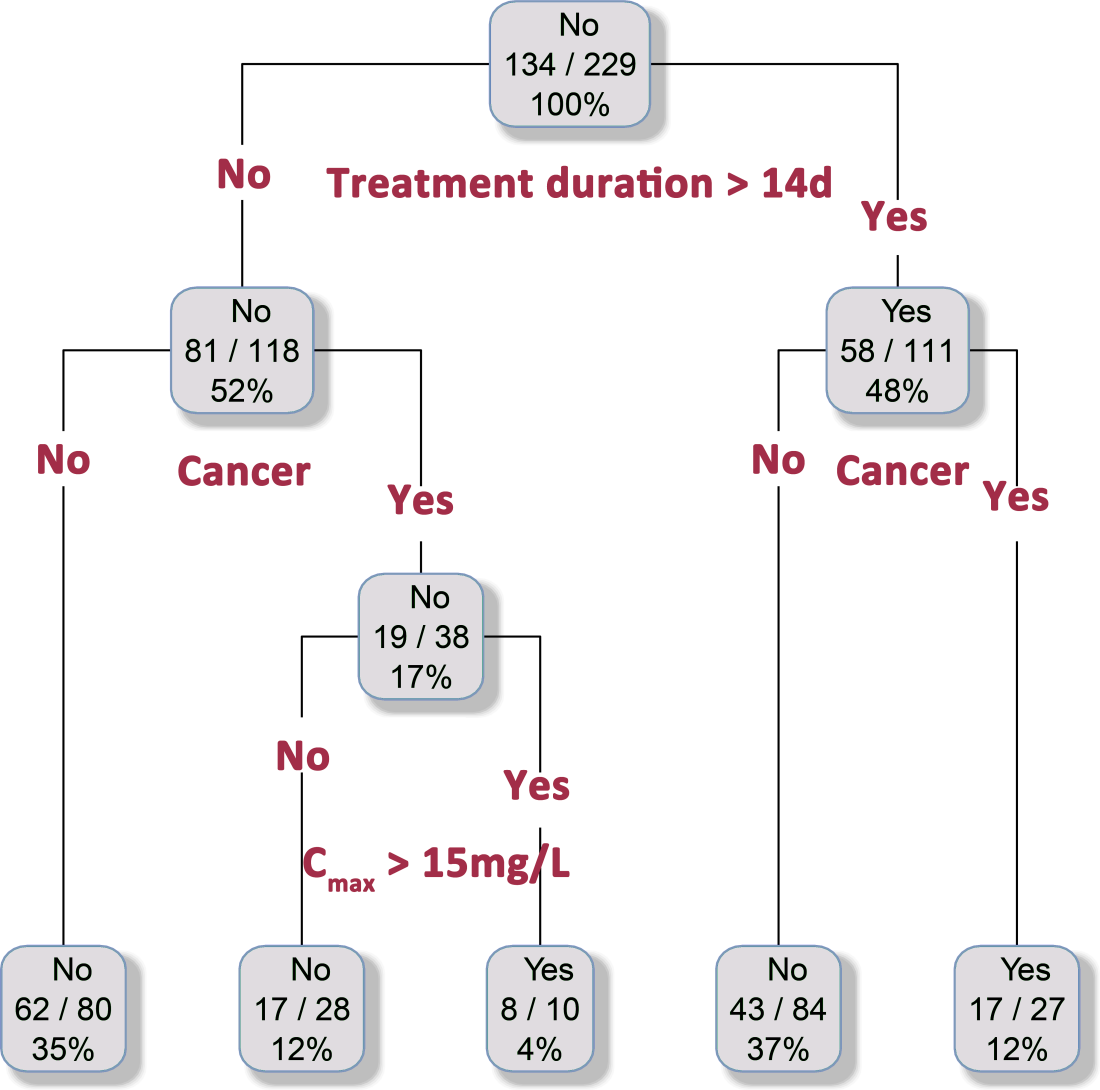
Decision tree model for predicting linezolid-associated hematologic adverse events, incorporating the following variables: presence of cancer, trough plasma concentration (*C*_min_) > 7 mg/L, peak plasma concentration (*C*_max_) > 15 mg/L, single dose of linezolid >11 mg/kg of body weight, and treatment duration >14 days.

Subsequently, a decision tree analysis was performed incorporating the following variables: presence of cancer, *C*_min_ > 7 mg/L, *C*_max_ > 15 mg/L, single dose of linezolid >11 mg/kg of body weight, and linezolid treatment duration >14 days (Fig. 3). The model demonstrated a significantly elevated incidence of hematologic adverse events among patients receiving linezolid therapy for >14 days, with cancer patients exhibiting particularly heightened susceptibility. Furthermore, a *C*_max_ > 15 mg/L was similarly associated with an increased risk of hematologic toxicity, an effect markedly amplified in the oncologic population.

## DISCUSSION

This study represents the largest pharmacokinetic-pharmacodynamic analysis of linezolid in pediatric patients to date, encompassing more than 200 children. Our findings highlight a significantly higher incidence of overall hematologic adverse events in cancer patients compared to non-cancer patients (60.00% vs 36.59%). Cancer patients exhibited a severalfold increased risk of thrombocytopenia and leukopenia events, whereas previously healthy children experienced relatively lower rates (<10%), except for anemia events, which were prevalent in both groups (>25%). Notably, *C*_max_ > 15 mg/L and treatment durations exceeding 14 days were associated with more than double the risk of specific hematologic adverse events. Comparative analysis of dosing regimens revealed that a twice-daily 600 mg regimen was linked to a higher rate of anemia events compared to a thrice-daily 10 mg/kg of body weight regimen in patients with similar weight and total daily doses. Furthermore, elevated *C*_min_ (>7 mg/L) was significantly associated with a more than threefold increased risk of leukopenia and anemia events in cancer patients. These findings emphasize the necessity of careful monitoring and individualized dose optimization of linezolid in high-risk groups to improve the safety profile for pediatric patients.

Consistent with prior research (12), previously healthy children exhibited low rates of leukopenia, neutropenia, and thrombocytopenia adverse events, each under 10%. However, in children who received anti-cancer chemotherapy, these risks increased dramatically, highlighting their heightened susceptibility to linezolid-induced hematologic toxicity. Previous retrospective studies have reported higher rates of thrombocytopenia (18%) and anemia (15%) (11), with a small-sample study noting a 53% incidence of all hematologic adverse events (8). These discrepancies may stem from variations in study designs and demographics, and our cohort may provide a more representative picture of the general pediatric population. Factors such as residual myelosuppression from anti-cancer chemotherapy or severe infections in immunocompromised children could contribute to these rates, potentially exacerbating myelosuppression (23, 24). Nonetheless, these real-world data underscore the high risk of hematologic impact in children who received anti-cancer chemotherapy, warranting further research into the interactions between linezolid, anti-cancer chemotherapy, and infection severity.

Anemia was the most common hematologic adverse event in our pediatric study population. Adult studies have identified the duration of linezolid treatment as the primary risk factor for anemia, with a median onset of 10–15 days post-initiation (25, 26). While pediatric data on the duration-anemia relationship are limited, a 15% anemia incidence was observed in studies with a median treatment duration of 8 days (11), and children with pre-existing anemia were at a higher risk of developing severe anemia following linezolid treatment (27). In our cohort, anemia onset was typically early, with a median of 3 days, aligning with peak infection severity. Anemia at this stage may be exacerbated by the infection itself and iatrogenic blood loss from frequent testing (28, 29). Although medical procedures and severe infections may contribute to anemia, the high incidence relative to the low proportion of patients with severe infections (30) suggests that linezolid-related anemia in children requires vigilance, with monitoring recommended around 3 days post-initiation. For other adverse events like leukopenia, neutropenia, and thrombocytopenia, onset may be later in patients without anti-cancer chemotherapy, but monitoring should begin approximately 1 week after starting the medication. Our findings align with previous research showing that the incidence of hematologic adverse events increases with the duration of linezolid treatment (14), likely due to cumulative drug-induced organ injury. The potential for drug accumulation and subsequent rise in adverse events with long-term linezolid use remains unclear in the absence of large-sample, real-world studies (31). However, continuous surveillance for adverse events during extended linezolid therapy is essential.

Additionally, our study suggested that, at equivalent daily dosages, a twice-daily linezolid regimen results in higher individual doses compared to a thrice-daily regimen, leading to increased *C*_max_ and potentially elevating the risk of adverse events. Further analysis indicated that administering a dose of ≥11 mg/kg of body weight per dose (above the standard pediatric dose of 10 mg/kg of body weight per dose) also yields a higher *C*_max_. Although the AUC_ss,24h_ and *C*_min_ were similar, this higher dosage correlated with an increased incidence of hematologic adverse events. Moreover, our study highlights that a *C*_max_ above 15 mg/L is associated with a heightened risk of hematologic adverse events. While previous research has linked high *C*_min_ and AUC_ss,24h_ of linezolid to increased adverse event risks (10, 11), our findings suggest that high *C*_max_ values may also contribute to hematologic toxicity. Future studies should explore the impact of brief, high-dose linezolid exposure on pediatric bone marrow hematopoiesis and mitochondrial function. Research is also warranted to assess whether increasing the frequency of linezolid administration from twice to three times daily in adolescents aged 12 and above, at equivalent daily doses, could mitigate hematologic toxicity risks. These findings also suggest that maintaining stable linezolid blood concentrations may help reduce the risk of hematologic toxicity. Adult studies show that adherence to instructions results in only about half of patients reaching target linezolid trough concentrations (32), and significant overexposure in elderly patients has been reported (20), making TDM recommended for all adults on linezolid therapy (33). Currently, no pediatric guidelines for TDM of linezolid exist, but achieving target drug exposure with standard dosing in children is also challenging, and the incidence of drug-related adverse events in children, especially those with hematologic abnormalities (27), appears higher. Therefore, this emphasizes the need for diligent monitoring of adverse events and drug concentrations, alongside precise dose optimization, to enhance the safety profile of linezolid in pediatric patients, particularly those at high risk.

### Limitations

This study has several limitations that warrant consideration. First, the analysis did not account for infection severity, which could influence hematologic parameters. However, the large sample size likely minimized the impact of this factor. Second, some children received concurrent medications that might have affected blood markers and the incidence of adverse events. Importantly, no patients received serotonergic drugs or monoamine oxidase inhibitors. A small subset received medications with potential pharmacokinetic interactions with linezolid (e.g., rifampicin, clarithromycin, cyclosporine, and omeprazole). However, in our study, individual pharmacokinetic parameters such as *C*_max_ and *C*_min_ were estimated using the MAP Bayesian approach, which integrates each patient’s measured concentrations with the population pharmacokinetic model of linezolid. This method allows for individualized predictions that inherently account for any unmeasured variability, including potential drug-drug interactions. Third, in patients with malignant diseases treated with linezolid, the myelosuppressive effects from anti-cancer chemotherapy could lead to an overestimation of hematologic adverse event rates. Therefore, caution is needed when interpreting comparative results between groups as presented in Table S1 and Fig. 1. Nonetheless, this limitation does not affect the analysis within the non-cancer group or the analysis of differences related to linezolid concentration, duration, dosage, and age within the overall population and the cancer group. Our study included a diverse cohort of hospitalized children receiving linezolid for various infections, including those receiving and who had received anti-cancer chemotherapy, providing a real-world perspective on its pediatric use. Future studies should include a control group treated with antibiotics that minimally affect peripheral blood cell counts to balance the effects of anti-cancer chemotherapy.

### Conclusion

Anemia represents a significant hematologic adverse event associated with linezolid therapy in pediatric patients, with the presence of cancer further amplifying the risk of hematologic toxicity. Additionally, a twice-daily dosing regimen, prolonged treatment durations, supratherapeutic peak concentrations (>15 mg/L), and trough levels (>7 mg/L in cancer patients) are correlated with increased hematologic toxicity. These observations highlight the critical need for diligent monitoring and careful dose optimization to enhance the safety of linezolid use in these vulnerable populations.

## SUPPLEMENTAL MATERIAL

Supplemental material aac.00294-25-s0001.docx Tables S1, S2, and S4 to S11; Fig. S1 to S3.

Table S3 aac.00294-25-s0002.xlsx PK parameters.

ASM does not own the copyrights to Supplemental Material that may be linked to, or accessed through, an article. The authors have granted ASM a non-exclusive, world-wide license to publish the Supplemental Material files. Please contact the corresponding author directly for reuse.
