# Supplementary material for: Pharmacokinetics and hematologic toxicity of linezolid in children: a prospective, two-center cohort study
Source: Antimicrob Agents Chemother. 2025 Jul 23;69(9):e00294-25. doi: 10.1128/aac.00294-25 (PMC12406672; doi:10.1128/aac.00294-25)
Supplement: Supplemental material — Tables S1, S2, and S4 to S11; Fig. S1 to S3. [file aac.00294-25-s0001.docx]

**Table S1. Hematologic adverse events of linezolid in children.**

| **Adverse events** | **All patients**  **(n = 229)** | **Non-cancer**  **(n = 164)** | **Cancer**  **(n = 65)** | ***P-*value** |
| --- | --- | --- | --- | --- |
| **All hematologic events, n (%)** | 99 (43.23) | 60 (36.59) | 39 (60.00) | 0.001 |
| Leukopenia events | 21 (9.17) | 2 (1.22) | 19 (29.23) | < 0.001 |
| Neutropenia events | 29 (12.66) | 16 (9.76) | 13 (20.00) | 0.036 |
| Anemia events | 66 (28.82) | 44 (26.83) | 22 (33.85) | 0.291 |
| Thrombocytopenia events | 24 (10.48) | 7 (4.27) | 17 (26.15) | < 0.001 |
| **Grade of leukopenia, n (%)** |  |  |  | < 0.001 |
| Grade 1 | 14 (6.11) | 2 (1.22) | 12 (18.46) |  |
| Grade 2-4 | 7 (3.06) | 0 (0.00) | 7 (10.77) |  |
| **Grade of neutropenia, n (%)** |  |  |  | 0.035 |
| Grade 1 | 13 (5.68) | 9 (5.49) | 4 (6.15) |  |
| Grade 2-4 | 16 (6.99) | 7 (4.27) | 9 (13.85) |  |
| **Grade of anemia, n (%)** |  |  |  | 0.376 |
| Grade 1 | 29 (12.66) | 21 (12.80) | 8 (12.31) |  |
| Grade 2-4 | 37 (16.16) | 23 (14.02) | 14 (21.54) |  |
| **Grade of thrombocytopenia, n (%)** |  |  |  |  |
| Grade 1 | 10 (4.37) | 6 (3.66) | 4 (6.15) | < 0.001 |
| Grade 2-4 | 14 (6.11) | 1 (0.61) | 13 (20.00) |  |
| **Time to adverse events (d), median (IQR)** |  |  |  |  |
| Time to leukopenia events | 4.00 (1.00-10.00) | 5.50 (3.25-7.75) | 4.00 (1.50-10.00) | 0.760 |
| Time to neutropenia events | 9.00 (3.00-16.00) | 10.50 (3.75-15.50) | 7.00 (2.00-16.00) | 0.468 |
| Time to anemia events | 3.00 (1.00-7.00) | 3.00 (1.00-6.00) | 3.00 (2.00-8.50) | 0.572 |
| Time to thrombocytopenia events | 5.00 (2.00-13.25) | 16.00 (4.50-24.00) | 2.00 (1.00-12.00) | 0.013 |

IQR: interquartile range.

**Table S2. Multivariable Cox regression analysis of all patients with stepwise covariate inclusion.**

| **Variables, HR (95% CI)** | **Leukopenia events** | **Neutropenia events** | **Anemia events** | **Thrombocytopenia events** |
| --- | --- | --- | --- | --- |
| **Adjusted age and gender** | | | | |
| Non-cancer | 1 | 1 | 1 | 1 |
| Cancer | 31.30 (7.08, 138.48) | 3.74 (1.70, 8.21) | 1.28 (0.75, 2.21) | 8.30 (3.31, 20.83) |
| **Adjusted age, gender, BMI and baseline blood cell count^a^** | | | | |
| Non-cancer | 1 | 1 | 1 | 1 |
| Cancer | 18.74 (3.84, 91.64) | 3.02 (1.24, 7.39) | 1.46 (0.75, 2.85) | 9.99 (2.99, 33.32) |
| **Adjusted age, gender, BMI, baseline blood cell count, site of infection and pathogen confirmed^b^** | | | | |
| Non-cancer | 1 | 1 | 1 | 1 |
| Cancer | 20.29 (3.98, 103.38) | 2.60 (1.00, 6.77) | 1.34 (0.65, 2.79) | 7.08 (2.19, 22.92) |

HR: Hazard Ratios; CI: Confidence interval; BMI: Body Mass Index;

^a^: declined or not‌; ^b^: with or without confirmed pathogen.

**Table S4. Incidences of linezolid-associated adverse events stratified by** **C_max_ of linezolid in blood.**

| **Adverse events, n (%)** | **C_max_, mg/L** | | ***P-*value** |
| --- | --- | --- | --- |
|  | **≤ 15 (n = 182)** | **> 15 (n = 47)** |  |
| **Leukopenia events** | 13 (7.14) | 8 (17.02) | 0.036 |
| **Neutropenia events** | 25 (13.74) | 4 (8.51) | 0.337 |
| **Anemia events** | 43 (23.63) | 18 (38.30) | 0.043 |
| **Thrombocytopenia events** | 15 (8.24) | 9 (19.15) | 0.030 |
| **All hematologic events** | 79 (43.41) | 20 (42.55) | 0.916 |

C_max_: peak linezolid blood concentration.

**Table S5. Incidences of linezolid-associated adverse events stratified by C_min_ of linezolid in blood.**

| **Adverse events, n (%)** | **C_min_, mg/L** | | ***P-*value** |
| --- | --- | --- | --- |
|  | **≤ 7** | **> 7** |  |
| **All patients** | **212** | **17** |  |
| Leukopenia events | 18 (8.49) | 3 (17.65) | 0.208 |
| Neutropenia events | 28 (13.21) | 1 (5.88) | 0.382 |
| Anemia events | 54 (25.47) | 7 (41.18) | 0.159 |
| Thrombocytopenia events | 21 (9.91) | 3 (17.65) | 0.316 |
| All hematological events | 93 (43.87) | 6 (35.29) | 0.492 |
| **Non-cancer group** | **152** | **12** |  |
| Leukopenia events | 2 (1.32) | 0 (0.00) | 0.689 |
| Neutropenia events | 16 (10.53) | 0 (0.00) | 0.237 |
| Anemia events | 35 (23.03) | 3 (25.00) | 0.876 |
| Thrombocytopenia events | 6 (3.95) | 1 (8.33) | 0.469 |
| **Cancer group** | **60** | **5** |  |
| Leukopenia events | 16 (26.67) | 3 (60.00) | 0.115 |
| Neutropenia events | 12 (20.00) | 1 (20.00) | 1.000 |
| Anemia events | 19 (31.67) | 4 (80.00) | 0.030 |
| Thrombocytopenia events | 15 (25.00) | 2 (40.00) | 0.463 |

C_min_: trough linezolid blood concentration.

**Table S6. Incidences of linezolid-associated hematologic adverse events stratified by each dose of linezolid.**

| **Characteristic** | **Each dose, mg/kg** | | ***P-*value** |
| --- | --- | --- | --- |
|  | **< 11 (n = 210)** | **≥ 11 (n = 19)** |  |
| **C_min_ (mg/L), median (IQR)** | 2.60 (1.43-4.07) | 2.63 (1.36-3.68) | 0.883 |
| **C_max_ (mg/L), median (IQR)** | 11.66 (9.34-13.90) | 16.33 (14.69-20.78) | < 0.001 |
| **AUC_ss,24h_ (mg‧h/L), median (IQR)** | 252.44 (205.13-291.93) | 286.02 (248.31-349.63) | 0.255 |
| **Adverse events, n (%)** |  |  |  |
| Leukopenia events | 17 (8.10) | 4 (21.05) | 0.061 |
| Neutropenia events | 27 (12.86) | 2 (10.53) | 0.770 |
| Anemia events | 51 (24.29) | 10 (52.63) | 0.007 |
| Thrombocytopenia events | 19 (9.05) | 5 (26.32) | 0.019 |
| All hematologic events | 88 (41.90) | 11 (57.89) | 0.178 |

C_min_: trough linezolid blood concentration; IQR: interquartile range; C_max_: peak linezolid blood concentration; AUC_ss,24h_: the area under the concentration curve across 24 hours at steady state of linezolid.

**Table S7. Incidences of linezolid-associated hematologic adverse events stratified by daily dose of linezolid.**

| **Adverse events, n (%)** | **Daily dose (mg/kg)** | | ***P-*value** |
| --- | --- | --- | --- |
|  | **≤ 30 (n = 190)** | **> 30 (n = 39)** |  |
| **Leukopenia events** | 17 (8.95) | 4 (10.26) | 0.796 |
| **Neutropenia events** | 23 (12.11) | 6 (15.38) | 0.575 |
| **Anemia events** | 51 (26.84) | 15 (38.46) | 0.144 |
| **Thrombocytopenia events** | 18 (9.47) | 6 (15.38) | 0.272 |
| **All hematologic events** | 78 (41.05) | 21 (53.85) | 0.142 |

**Table S8. Incidences of linezolid-associated hematologic adverse events stratified by duration of linezolid therapy.**

| **Adverse events, n (%)** | **Duration, d** | | | ***P-*value** | **Duration, d** | | ***P-*value** | **Duration, d** | | ***P-*value** |
| --- | --- | --- | --- | --- | --- | --- | --- | --- | --- | --- |
|  | **≤ 14** | **> 14, ≤ 28** | **> 28** |  | **≤ 14** | **> 14** |  | **≤ 28** | **> 28** |  |
| **All patients** | **128** | **58** | **43** |  | **118** | **111** |  | **184** | **45** |  |
| Leukopenia events | 10 (7.81) | 7 (12.07) | 4 (9.30) | 0.647 | 10 (8.47) | 11 (9.91) | 0.707 | 16 (8.70) | 5 (11.11) | 0.615 |
| Neutropenia events | 10 (7.81) | 9 (15.52) | 10 (23.26) | 0.023 | 10 (8.47) | 19 (17.12) | 0.049 | 19 (10.33) | 10 (22.22) | 0.031 |
| Anemia events | 25 (19.53) | 20 (34.48) | 16 (37.21) | 0.022 | 21 (17.80) | 40 (36.04) | 0.002 | 44 (23.91) | 17 (37.78) | 0.059 |
| Thrombocytopenia events | 12 (9.38) | 6 (10.34) | 6 (13.95) | 0.697 | 11 (9.32) | 13 (11.71) | 0.555 | 18 (9.78) | 6 (13.33) | 0.486 |
| All hematologic events | 49 (38.28) | 32 (55.17) | 26 (60.47) | 0.014 | 45 (38.14) | 62 (55.86) | 0.007 | 79 (42.93) | 28 (62.22) | 0.020 |
| **Non-cancer group** | **88** | **41** | **35** |  | **80** | **84** |  | **128** | **36** |  |
| Leukopenia events | 2 (2.27) | 0 (0.00) | 0 (0.00) | 0.417 | 2 (2.50) | 0 (0.00) | 0.145 | 2 (1.56) | 0 (0.00) | 0.450 |
| Neutropenia events | 5 (5.68) | 5 (12.20) | 6 (17.14) | 0.128 | 5 (6.25) | 11 (13.10) | 0.140 | 10 (7.81) | 6 (16.67) | 0.114 |
| Anemia events | 10 (11.36) | 13 (31.71) | 15 (42.86) | < 0.001 | 8 (10.00) | 30 (35.71) | < 0.001 | 22 (17.19) | 16 (44.44) | < 0.001 |
| Thrombocytopenia events | 3 (3.41) | 1 (2.44) | 3 (8.57) | 0.353 | 3 (3.75) | 4 (4.76) | 0.749 | 4 (3.12) | 3 (8.33) | 0.172 |
| All hematologic events | 22 (25.00) | 20 (48.78) | 21 (60.00) | < 0.001 | 20 (25.00) | 43 (51.19) | < 0.001 | 41 (32.03) | 22 (61.11) | 0.002 |
| **Cancer group** | **40** | **17** | **8** |  | **38** | **27** |  | **56** | **9** |  |
| Leukopenia events | 8 (20.00) | 7 (41.18) | 4 (50.00) | 0.106 | 8 (21.05) | 11 (40.74) | 0.085 | 14 (25.00) | 5 (55.56) | 0.061 |
| Neutropenia events | 5 (12.50) | 4 (23.53) | 4 (50.00) | 0.049 | 5 (13.16) | 8 (29.63) | 0.102 | 9 (16.07) | 4 (44.44) | 0.048 |
| Anemia events | 15 (37.50) | 7 (41.18) | 1 (12.50) | 0.340 | 13 (34.21) | 10 (37.04) | 0.814 | 22 (39.29) | 1 (11.11) | 0.101 |
| Thrombocytopenia events | 9 (22.50) | 5 (29.41) | 3 (37.50) | 0.637 | 8 (21.05) | 9 (33.33) | 0.267 | 14 (25.00) | 3 (33.33) | 0.597 |
| All hematologic events | 27 (67.50) | 12 (70.59) | 5 (62.50) | 0.921 | 25 (65.79) | 19 (70.37) | 0.697 | 38 (67.86) | 6 (66.67) | 0.943 |

**Table S9. Incidences of linezolid-associated hematologic adverse events stratified by age.**

| **Adverse events** | **Age, y** | | | | ***P-*value** |
| --- | --- | --- | --- | --- | --- |
|  | **< 3** | **≥ 3, < 7** | **≥ 7, < 12** | **≥ 12** |  |
| **No. of patients** | 88 | 48 | 67 | 26 |  |
| **Time to leukopenia events, d, median (IQR)** | 9.00 (2.00-13.00) | 2.50 (1.00-21.25) | 5.00 (2.00-6.00) | 3.00 (3.00-7.00) | 0.641 |
| **Leukopenia events, n (%)** | 5 (5.68) | 6 (12.77) | 5 (7.46) | 5 (19.23) | 0.150 |
| **Time to neutropenia events, d, median (IQR)** | 11.00 (4.00-16.00) | 28.00 (15.00-28.50) | 4.50 (2.25-6.75) | 9.00 (8.00-12.00) | 0.905 |
| **Neutropenia events, n (%)** | 17 (19.32) | 3 (6.25) | 6 (8.96) | 3 (11.54) | 0.104 |
| **Time to anemia events, d, median (IQR)** | 3.00 (1.00-10.00) | 2.00 (1.00-3.50) | 3.50 (2.00-7.50) | 4.50 (1.75-14.50) | 0.440 |
| **Anemia events, n (%)** | 29 (32.95) | 11 (22.92) | 14 (20.90) | 12 (46.15) | 0.060 |
| **Time to thrombocytopenia events, d, median (IQR)** | 13.00 (4.00-23.00) | 2.00 (1.50-8.00) | 1.50 (1.00-2.00) | 6.50 (3.00-11.50) | 0.150 |
| **Thrombocytopenia events, n (%)** | 9 (10.23) | 3 (6.25) | 6 (8.96) | 6 (23.08) | 0.140 |

IQR: interquartile range.

**Table S10. Multivariable Cox regression analysis with stepwise covariate inclusion.**

| **Variables, HR (95% CI)** | **Leukopenia events** | **Neutropenia events** | **Anemia events** | **Thrombocyto-penia events** |
| --- | --- | --- | --- | --- |
| **Adjusted age and gender** | | | | |
| Non-cancer | 1 | 1 | 1 | 1 |
| Cancer | 31.30 (7.08, 138.48) | 3.74 (1.70, 8.21) | 1.28 (0.75, 2.21) | 8.30 (3.31, 20.83) |
| **Adjusted age, gender, BMI, baseline blood cell counts^a^ and C_max_^b^** | | | | |
| Non-cancer | 1 | 1 | 1 | 1 |
| Cancer | 16.70 (3.46, 80.60) | 3.15 (1.27, 7.80) | 1.46 (0.75, 2.84) | 8.77 (2.72, 28.34) |
| **Adjusted age, gender, BMI, baseline blood cell counts, C_min_^c^, C_max_ and treatment duration^d^** | | | | |
| Non-cancer | 1 | 1 | 1 | 1 |
| Cancer | 18.57 (3.67, 93.96) | 3.19 (1.25, 8.10) | 1.51 (0.78, 2.92) | 9.37 (2.72, 32.31) |

HR: Hazard Ratios; CI: Confidence interval; BMI: Body Mass Index; C_max_: peak linezolid blood concentration, C_min_: trough linezolid blood concentration.

^a^: declined or not‌; ^b^: ≤15 mg/L or >15 mg/L; ^c^: ≤7 mg/L or >7 mg/L; ^d^: ≤14 days or >14 days.

**Table S11. Multivariable Cox regression analysis of adverse advents between cancer and non-cancer groups across stratification variables.**

| **Variables, HR (95% CI)** | **Leukopenia events** | **Neutropenia events** | **Anemia events** | **Thrombocytopenia events** |
| --- | --- | --- | --- | --- |
| **C_min_^a^** | |  |  |  |
| **≤ 7 mg/L** | |  |  |  |
| Non-cancer | 1 | 1 | 1 | 1 |
| Cancer | 15.19 (3.04, 75.91) | 2.84 (1.10, 7.33) | 1.25 (0.63, 2.49) | 10.12 (2.73, 37.48) |
| **> 7 mg/L** | | | | |
| Non-cancer | 1 | 1 | 1 | 1 |
| Cancer | NA | NA | NA | NA |
| **C_max_^b^** |  |  |  |  |
| **≤ 15 mg/L** |  |  |  |  |
| Non-cancer | 1 | 1 | 1 | 1 |
| Cancer | 60.10 (5.05, 714.77) | 2.23 (0.77, 6.45) | 1.51 (0.65, 3.52) | 56.01 (8.32, 376.85) |
| **> 15 mg/L** |  |  |  |  |
| Non-cancer | 1 | 1 | 1 | 1 |
| Cancer | 10.67 (0.89, 127.40) | NA | 1.80 (0.46, 7.08) | 3.63 (0.55, 24.12) |
| **Duration of linezolid^c^** | |  |  |  |
| **≤ 14 d** |  |  |  |  |
| Non-cancer | 1 | 1 | 1 | 1 |
| Cancer | 1.42 (0.16, 12.28) | 3.76 (0.54, 25.92) | 4.40 (0.94, 20.58) | 2.36 (0.33, 16.96) |
| **> 14 d** |  |  |  |  |
| Non-cancer | 1 | 1 | 1 | 1 |
| Cancer | NA | 3.41 (1.08, 10.78) | 1.06 (0.46, 2.44) | 28.06 (4.20, 187.51) |

HR: Hazard Ratios; CI: Confidence interval; C_min_: Trough linezolid concentration; C_max_: Peak linezolid concentration.

^a^: Adjusted age, gender, Body Mass Index (BMI), Baseline blood cell counts (declined or not), C_max_ (≤ 15 mg/L or > 15 mg/L) and Duration of linezolid (≤ 14 days or > 14 days).

^b^: Adjusted age, gender, BMI, Baseline blood cell counts, C_min_ (≤ 7 mg/L or > 7 mg/L) and Duration of linezolid.

^c^: Adjusted age, gender, BMI, Baseline blood cell counts, C_max_ and C_min_.

NA: data not available. HRs could not be calculated due to insufficient case numbers for statistical analysis.

**244** children met the initial inclusion/exclusion criteria

**164** children

**13 excluded:**

**7** had no eligible samples for linezolid concentration testing

**3** lacked blood cell counts on or within 2 days before linezolid medication

**3** missed post-linezolid blood cell count exams

**Cancer group:**

**67** children with malignant solid tumors or hematological malignancies who had undergone or were undergoing chemotherapy within the month prior to linezolid administration

**Non-cancer group:**

**177** children who were previously healthy

**2 excluded:**

**2** had no eligible samples for linezolid concentration testing

**65** children

**Figure S1.** Study flowchart.


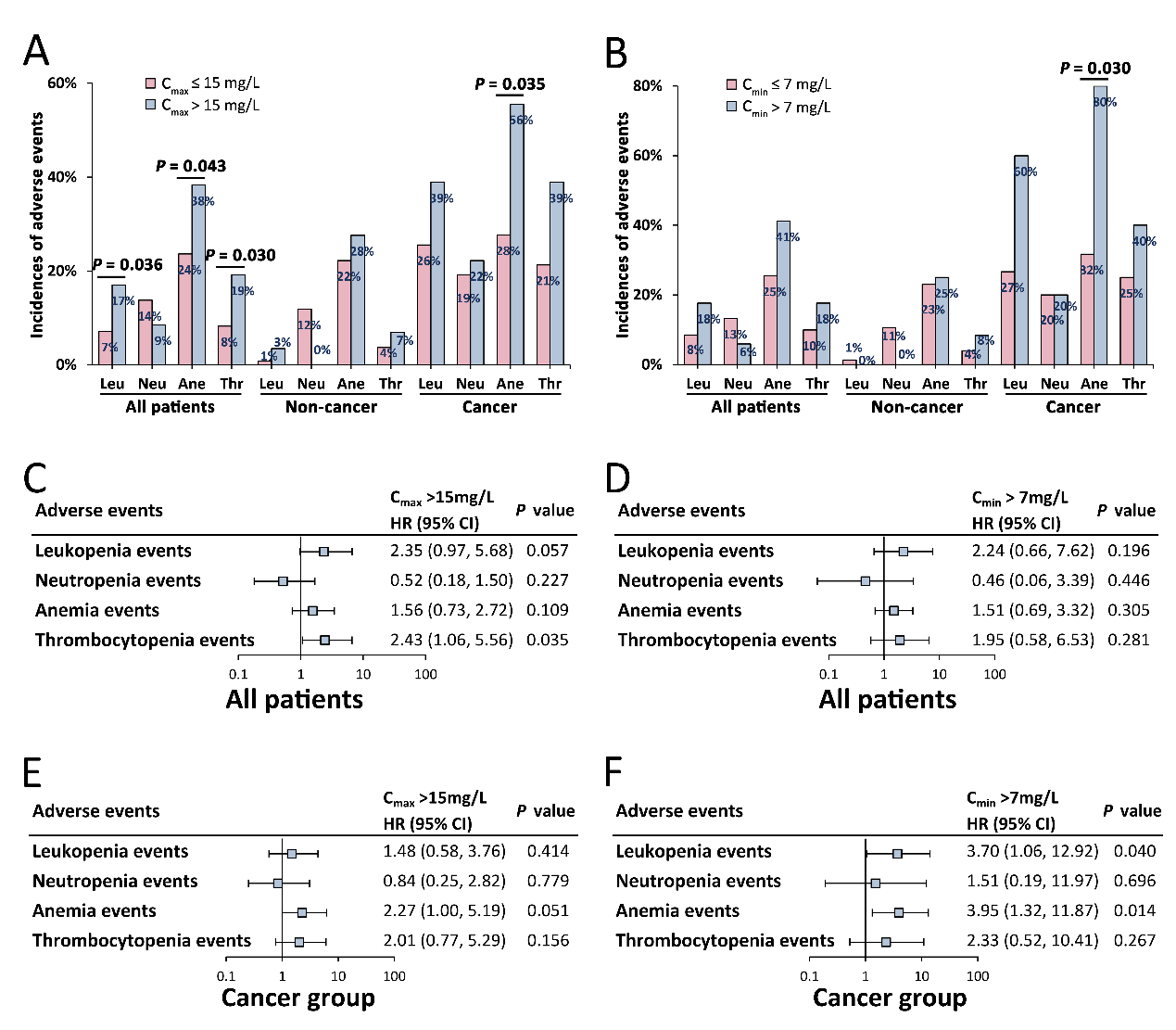


**Figure S2.** Linezolid-associated Hematologic Adverse Events Stratified by Linezolid Blood Concentrations. Patients with a C_max_ > 15 mg/L had higher incidences of leukopenia, anemia, and thrombocytopenia events. In cancer group, a C_max_ > 15 mg/L had increased incidences of anemia events, a trend also observed in patients with a C_min_ > 7 mg/L (**A, B**). Forest plots indicated that the risk of thrombocytopenia events was elevated in patients with a C_max_ > 15 mg/L; however, no significant difference was noted when stratified by a C_min_ of 7 mg/L in the overall patient population (**C, D**). In cancer group, those with a C_min_ > 7 mg/L had higher incidences of leukopenia and anemia events, yet no significant difference was observed when stratified by a C_max_ of 15 mg/L (**E, F**).

C_max_: peak linezolid blood concentration, C_min_: trough linezolid blood concentration, Leu: leukopenia adverse events, Neu: neutropenia adverse events, Ane: anemia adverse events; Thr: thrombocytopenia adverse events, HR: hazard ratio, CI: confidence interval.


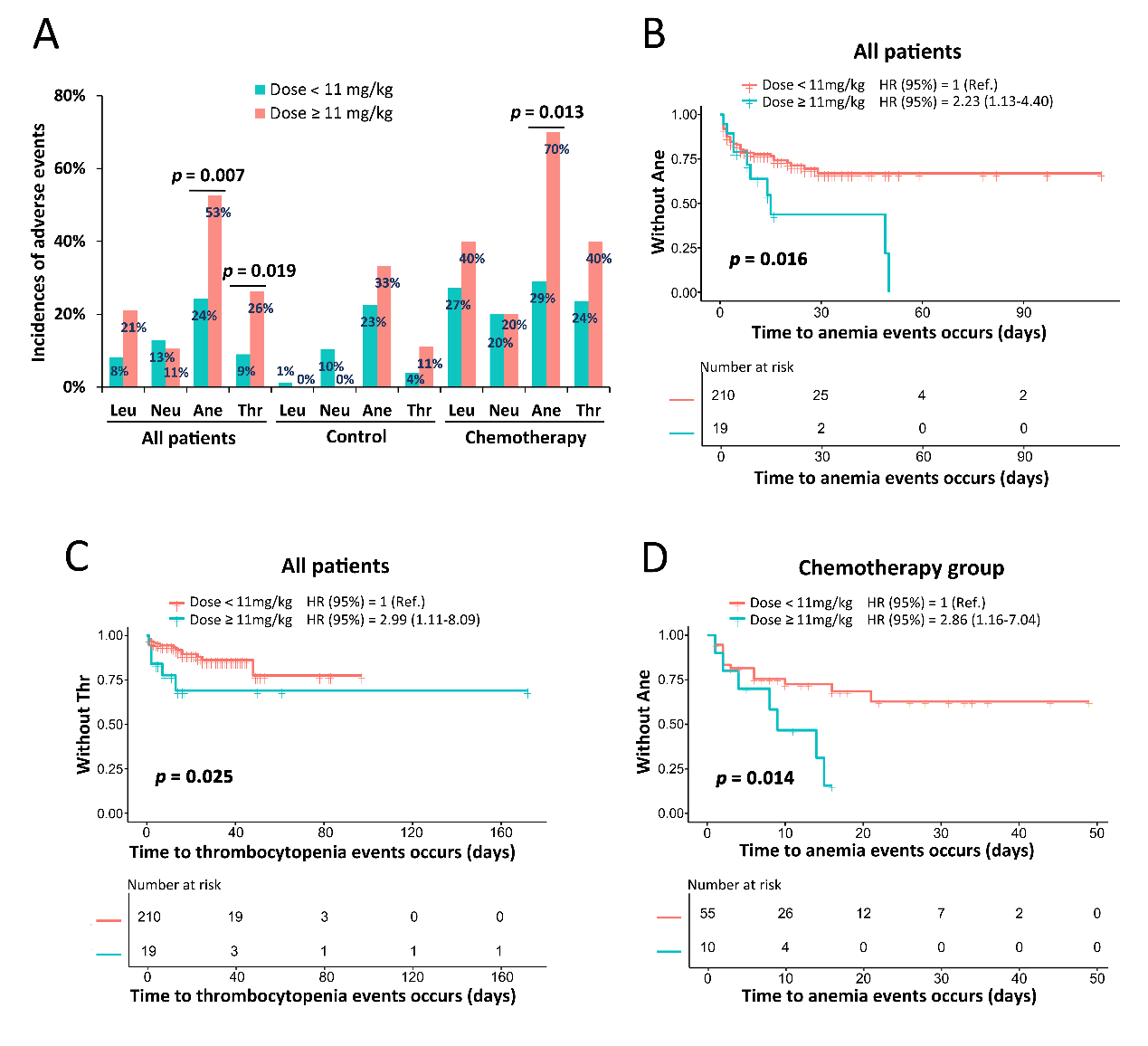


**Figure S3.** Incidences of linezolid-associated adverse events stratified by dose of linezolid. Patients with higher each dose of linezolid had high incidences of anemia events and thrombocytopenia events, but there was no difference in non-cancer group while in cancer group, higher incidence of thrombocytopenia events was observed (A). Kaplan-Meier curves show that patients with higher each dose of linezolid had greater risk of anemia events and thrombocytopenia events (B, C), also the risk of anemia events in chemotherapy group (D).

Leu: leukopenia adverse events, Neu: neutropenia adverse events, Ane: anemia adverse events, Thr: thrombocytopenia adverse events, HR: hazard ratio.
